# The Evaluation of Flow-Mediated Vasodilation in the Brachial Artery Correlates With Endothelial Dysfunction Evaluated by Nitric Oxide Synthase Metabolites in Marfan Syndrome Patients

**DOI:** 10.3389/fphys.2018.00965

**Published:** 2018-08-21

**Authors:** Oscar Lomelí, Israel Pérez-Torres, Ricardo Márquez, Sergio Críales, Ana M. Mejía, Claudia Chiney, Enrique Hernández-Lemus, Maria E. Soto

**Affiliations:** ^1^Department of Echocardiography, National Institute of Cardiology “Ignacio Chávez”, Mexico City, Mexico; ^2^Department of Pathology, National Institute of Cardiology “Ignacio Chávez”, Mexico City, Mexico; ^3^Department of Immunology, National Institute of Cardiology “Ignacio Chávez”, Mexico City, Mexico; ^4^Department of Computed Tomography, National Institute of Cardiology “Ignacio Chávez”, Mexico City, Mexico; ^5^Blood Bank, National Institute of Cardiology “Ignacio Chávez”, Mexico City, Mexico; ^6^Central Laboratory, National Institute of Cardiology “Ignacio Chávez”, Mexico City, Mexico; ^7^Computational Genomics Division, National Institute of Genomic Medicine, Mexico City, Mexico

**Keywords:** Marfan syndrome, flow-mediated vasodilation, endothelial dysfunction, inflammation, nitric oxide

## Abstract

Marfan syndrome (MS) is of the most common connective tissue disorders. Although most patients have mutations in the fibrillin-1 gene (FBN1) and more than 1,700 mutations have been described, there are no mutations in less than 10% of patients. Aortic dilation is the most important complication; it involves chronic inflammatory processes and endothelial dysfunction. Prospective study from March 2015 to January 2017, in a cohort of 32 patients of MS confirmed by Ghent criteria and 35 controls of both genders, with a median age of 26 years (18–56). Patients had no comorbidities such as diabetes, hypertension, and/or neoplasms. They were not being treated with statin, NSAIDs, calcium antagonists, oral nitrates, and/or beta-blockers during 7 days prior to the study and patients with smoking history in the last 4 years. Controls were matched by age and gender. We analyzed endothelial dysfunction by flow-mediated vasodilation in the brachial artery, determining the maximum peak flow in the reactive hyperemia phase with a Philips Envisor device with Doppler capability. Its correlation with serum levels of biological markers that could participate in endothelial dysfunction pathways such as ${\text{NO}}_{3}^{-}/{\text{NO}}_{2}^{-}$ ratio, ${\text{NO}}_{2}^{-}$, citrulline, TNFα, IL-1, IL-6, IL-10, IL-8, osteopontin, ICAM, VCAM, and ${\text{NO}}_{3}^{-}/{\text{NO}}_{2}^{-}$ was determined. Endothelial dysfunction was found in 21 MS patients (65%). The aortic annulus (AAo) was of 27 mm (22–40) and 24 mm (22–30) (*p* = 0.04) in MS patients with and without dysfunction. The level of ${\text{NO}}_{3}^{-}/{\text{NO}}_{2}^{-}$ ratio, was of 108.95 ± 12.05 nM/ml in controls vs. 170.04 ± 18.76 nM/ml in MS (*p* = 0.002), ${\text{NO}}_{2}^{-}$ was of 33.78 ± 3.41 vs. 43.95 ± 2.59 nM/ml (*p* = 0.03), citrulline 62.65 ± 3.46 vs. 72.81 ± 4.35 μMol/ml (*p* = 0.06). VCAM median was 39 pg/ml (0–86) vs. 32 pg/ml (11–66) (*p* = 0.03), respectively. The correlation of VCAM with triglycerides (TG) was of 0.62 (*p* = 0.005). There were no differences in TNFα, IL-1, IL-6, IL-8, IL-10, and osteopontin. MS endothelial dysfunction is related to aortic diameters, and increased levels of VCAM, L-citrulline and ${\text{NO}}_{3}^{-}/{\text{NO}}_{2}^{-}$ ratio, ${\text{NO}}_{2}^{-}$. VCAM-1 has a significant correlation with TG and could play a significant role in endothelial dysfunction.

## Introduction

Marfan syndrome (MS) is rare disease with a dominant autosomal hereditary pattern that has an overall incidence of 3/10,000. It is related to 1,700 mutations in the fibrillin-1 (FBN1) gene (Dietz, 2017). The FBN1 gene is 250 kb long; it is composed of 65 exons, and is located in chromosome 15q-21.1. From the mutational repertoire, up to 25% of mutations can be *de novo* mutations and about 10% have not been identified (Judge and Dietz, 2005). FBN1 is essential component of elastic and inelastic connective tissues. FBN1 helps in the transference of the hemodynamic burden and in the alignment of the fibers along the direction of parietal stress. Therefore, FBN1 is involved in the protective mechanisms that prevent over-distension of elastin, improving arterial elasticity. This can result in flow-mediated vasodilation (Eberth et al., 2009). The augmented activity of the TGFB signaling pathway may lead to elastic fiber disruption and to an increase in collagen reservoirs in MS (Yang H.H. et al., 2010).

The ascending aorta is commonly affected by dilation and/or dissection in this disease, that constitute the main causes of morbidity and mortality. Experimental studies in vascular hemodynamics in homozygotic mutant mice to mgR have shown mechanic alterations secondary to vascular structural changes that compensate for the lost elasticity, hence maintaining intravascular hemodynamic homeostasis (Eberth et al., 2009). Ultrasound measurements in MS patients have shown a delayed expansion and synchrony during systole in carotid arteries.

Endothelial dysfunction has been proposed as a mechanism accounting for aortic dilation (Takata et al., 2014). There is an increase in the inducible nitric oxide synthase (iNOS) in animal models (Yang H.H. et al., 2010) and other studies have described diminished levels of phosphorylated eNOS and augmented levels of iNOS. The increase in iNOS was associated with over-production of NO in SM patients. Excessive iNOS-driven NO production causes cellular damage via accumulation of peroxynitrites (ONOO^−^). Peroxynitrites are associated to the inflammatory pathway that is one of the main players in the formation of aortic aneurysms in MS patients (Soto et al., 2016a).

Increased vasodilation in MS may be related to other aspects besides NO availability. Murine model studies have shown participation of the cyclooxygenase pathway. Diminished levels of alpha 2 thromboxane, mild expression of type 1- cyclooxygenase and of an increase in type 2- cyclooxygenase also play an important role (Soto et al., 2018). These factors lead to an increase in I_2_ prostaglandin levels. These factors have as a consequence an overall diminution of the contraction of the thoracic aorta and to a severe compromise its structure and function (Chung et al., 2007b).

In addition, an animal model of MS, showing IL-6 deficiency, partially preserved the structure of the extracellular matrix, suggesting a role for IL-6 in the pathologic remodeling of the aortic wall (Ju et al., 2014). An increase in IL-6 in the adventitia has been found in the dissection site of human aneurisms (Doyle et al., 2012). A study done in IL-1β deficient mice, revealed diminished aneurism progression, low levels of cytokines and metalloprotease 9 (MMP9). These results point out that IL-1β may be a potential target for the treatment of aneurisms in the thoracic aorta (Johnston et al., 2014). Furthermore, IL-10 levels are significantly reduced in MS patients when compared to controls subjects (Kadoglou et al., 2012).

In spite of these findings in the aortic disease of MS patients, it remains unclear whether these mechanisms are the only ones associated with endothelial dysfunction. Abnormal response to flow mediated vasodilation could also be related to the mechano-transductional mechanisms (Wilson et al., 1999). The production and release of NO responds to biomechanical effects and therefore, the study of endothelial dysfunction by bidimensional ultrasound and Doppler sonography of the brachial artery might be useful for the assessment of flow-mediated vasodilation. Friction-force stimulus generated by sudden blood flow over the brachial artery might lead to endothelial NO release with a concomitant measurable vasodilation (Corretti et al., 2002). However, since NO production in serum is unlikely to happen, in the present study we evaluated serum levels of ${\text{NO}}_{3}^{-}/{\text{NO}}_{2}^{-}$ ratio, ${\text{NO}}_{2}^{-}$ and citrulline, as alternative biomarkers to detect endothelial dysfunction and their correlation to flow-mediated vasodilation.

## Materials and Methods

This is a comparative and prospective cohort observational study that took place between the years 2015–2016.

### Population Under Study

We included MS patients, evaluated by a rheumatologist using Ghent criteria (Loeys et al., 2010). Male and female subjects with an age above 18 years, without chronic or acute disease, or neoplasms were included. All individuals had a 7 day wash-out of statin, NSAIDs, calcium antagonists, oral nitrates and beta blockers, with negative serology for HCV, HBV, HIV, syphilis, and Chagas disease. Controls were paired by age and gender, from blood bank volunteers. All participating healthy subject were given a clinical record and were subjected to physical exploration to determine the absence of clinical MS criteria. Controls were also analyzed by the same serologic tests to discard infection.

### Exclusion Criteria

For MS cases, patients that had not suspended statins, NSAIDs, calcium antagonists, oral nitrites, and beta-blockers 7 days prior to sampling or ultrasound; subjects with a previous aortic surgery or with associated comorbidities such as diabetes, thyroid disease, arterial hypertension, coronary disease, peripheral arterial disease, angioplasty of the upper limbs, cervical sympathectomy, smoking or that were unwilling to sign informed consent were excluded.

For controls, exclusion criteria were: individuals with first and second- degree familiar relationships with MS or similar diseases or thoracic aneurism related maladies. Pregnant women and those in menopause or menstruating were also excluded.

### Laboratory Tests

HDL and LDL lipoprotein, triglycerides (TG), and serum glucose were tested. Sample obtainment and flow-mediated vasodilation were performed the same day with a maximum delay of 2 h between sample obtainment and cabinet studies to evaluate endothelial dysfunction. Both studies were done under fasting conditions. For the determination of ${\text{NO}}_{3}^{-}/{\text{NO}}_{2}^{-}$ ratio, ${\text{NO}}_{2}^{-}$ and citrulline it was required that both, patients and controls did not perform physical exercise during 24 h previous to sample obtainment. Consumption of flavonoids, theobromine, some fruits and vegetables, olive oil, fish oil, beef or pork, red wine, chocolate, coffee, cocoa, soy, or tea was avoided by all included subjects.

#### Ethics Statement

The study was carried out according to the international ethical standards and the General Health Law, as well as according to the Helsinki Declaration, modified at the Congress of Tokyo, Japan. Also, this protocol (PT 15-15) was approved by the local ethical committee. All of the patients and controls read and signed an informed consent form.

### Flow-Mediated Vasodilation

**FIGURE 1:**
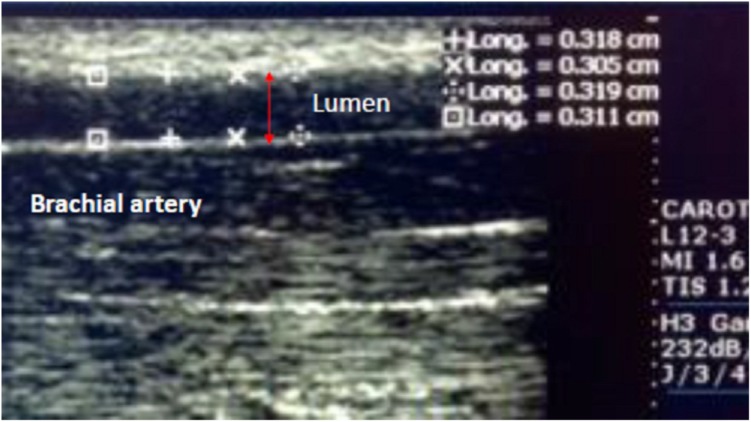
Shows a segment of the artery located at the anterior and posterior portions of the intima. This image was obtained by using grayscale bidimensional ultrasound over a longitudinal plane to the artery.

Flow-mediated vasodilation was determined by brachial artery ultrasound sonography. The study took place in the radiology department at our institution, in a temperature controlled room (21–23°C), under fasting conditions including both liquids and solids, for a minimum of 8 h, and after a 7 day wash out of the already mentioned anti-inflammatory drugs. Four hours previous to the study, subjects had not ingested caffeine, c vitamin, smoked or chewed tobacco and they had restrained from physical exercise. Women were not studied during menstruation. Ultrasound studies were performed with a Phillips Envisor device that is able to measure colored and pulsatile Doppler (to determine the maximum peak of flow in the reactive hyperemia phase). The device was synchronized with a monitor that is able to register cardiac frequency and a high frequency transducer (7–12 MHz) was used. The subject under study was placed in supine decubit position with the arm in a comfortable position to allow for brachial artery detection. The subjects remained at rest for 10 min prior to basal image acquisition. A clear segment of the artery was found by locating the anterior and posterior portions of the intima and by using grayscale bidimensional ultrasound over a longitudinal plane to the artery (5 cm) (see **Figure 1**).

To create a stimulus similar to that producing flow-like vasodilation, a sphygmomanometer was used, placing it over the antecubital pit (or below) to take a basal image of the artery. Next, the sphygmomanometer was inflated to at least 50 mmHg over the systolic pressure of the patient for 5 min. A continuous image was recorded for 30 s prior to the beginning of deflation and finished 2 min after deflation completion (in total, 150 s were recorded). Continuous velocity recording of this period (deflation or reactive hyperemia) is shown in **Table 4**. Images taken at 60 and 120 s after deflation were analyzed. The tele-diastolic period, and the peak of the R wave (four measurements of the lumen of the vessel were made and the average was taken for the analysis of fractional changes) were taken into account for the measurements. The percentage rate of change in diameter of the brachial artery was calculated as follows (**Table 2**):

(Maximum diastolic diameter−Basal diastolic diameter)×100.

### Basal Diastolic Diameter

An increase greater or equal to 10% in the size of the diastolic diameter of the brachial artery as related to the basal image, was taken as evidence of response to the maneuver, 60 s into the reactive hyperemia period (after sphygmomanometer deflation) with the subjects being their own controls.

### Sample Size

Since it was an exploratory study, there is no previous evidence to support for statistical sample size calculations. The number of MS patients entering the National Institute of Cardiology “Ignacio Cháve” (NIC) varies from 15 to 2 per year. Age- and gender- paired healthy controls were included for comparison. Average flow velocity (AFV) with a fractional change of less than 10% in the diastolic diameter of the brachial artery is considered as a sign of true damage when its change is around 0.02. In contrast, for a healthy population, the fractional change in AFV with a change in diameter is greater than 10%. To this end, we recruited 33 cases and 33 controls adjusted for losses. The model is as follows:

$${\text{H}}_{\text{o}}:p=\mathrm{po versus p}\neq \text{po}$$

A=0.051−β=0.80

$$P_{\text{o}}=0.10$$

$$P_{1}=0.02$$

$$n=\frac{\mathrm{poqo}{\left[Z_{1-\alpha /2}+Z_{1-\beta }\sqrt{\frac{p_{1}q_{1}}{\mathrm{poqo}}}\right]}^{2}}{p_{1}-\mathrm{po}}$$

$$n=\frac{0.10(0.90){\left[Z_{0.975}+Z_{0.80}\sqrt{\frac{0.02{\left(0.98\right)}_{1}}{0.10(0.90)}}\right]}^{2}}{{\left(0.08\right)}^{2}}$$

### Variable Specification

#### Endothelial Dysfunction

Endothelial dysfunction was assessed by brachial artery ultrasound and defined as a response lower than 10% in the enlargement of the diastolic diameter (as compared to basal measurement) in the 60 s of the reactive hyperemia (posterior to sphygmomanometer deflation).

#### Ascending Aorta Dilation

Ascending aortic dilation was defined as an absolute value greater than 34 mm for males and greater than 31 mm for females.

#### Citrulline Determination

Hundred microliter of serum were incubated for 30 min at 37°C after addition of 50 μL urease 12 mg/mL and of 3 mL of chromic mixture. The chromic mixture consisted of 25% H_2_SO_4_, 20% H_3_PO_4_, 9.24 μM FeCl_3_⋅6H_2_O, 0.125% 2,3-butanedione monoxime, and 0.0075% thiosemicarbazide mixed by vortex and incubated at 100°C for 5 min. The samples were cooled to room temperature and the color developed was measured at 530 nm. The calibration curve was made with a standard solution of L-citrulline 1 μmol/L from Sigma-Aldrich (St. Louis, MO, United States) (Pérez-Torres et al., 2016).

#### Nitrate and Nitrite Quantification

For ${\text{NO}}_{3}^{-}/{\text{NO}}_{2}^{-}$ quantification, 100 μl of previously de-proteinized serum were incubated with 50 μl Cu-Cd for 30 min. The mixture was centrifuged at 850 *g*, at room temperature for 5 min. and the supernatant was recovered and incubated in the presence 100 μl of sulfanilamide 1% and 100 μl of *N*-naphthyl-ethyldiamine 0.1%. The total volume was adjusted to 1 ml (Pérez-Torres et al., 2016). For ${\text{NO}}_{2}^{-}$, 100 μl of previously de-proteinized serum were added to 100 μl of sulfanilamide 1% and 100 μl of *N*-naphthyl-ethyldiamine 0.1% and the total volume was adjusted to 1 ml. Both quantifications were measured at 540 nm. The calibration curve was obtained using a solution of KNO_2_ at concentration ranging from 5 to 0.156 nM.

#### Inflammatory Interleukins

Levels of human inflammatory interleukin mediators were measured with Quantikine ELISA assays (R&D systems, Minneapolis, MN, United States), using specific kits to TNFα (Cat DTA00C), IL-1β (Cat. DLB50), IL-6 (Cat. D6050), IL-8 (Cat. D8000C), IL-10 (Cat. D1000B), Osteopontin (Cat. DOST00), VCAM (Cat. DVC00), and ICAM (DCD540). Briefly, in a 96 well polystyrene microplate coated with a primary anti-cytokine monoclonal antibody, 200 μl of standard (reference curve), samples or control were added to each well, in duplicates. The plate was incubated for 2 h at room temperature. After that, each well was aspirated and washed with 300 μl of wash buffer. This step was repeated three times, and then the plate was inverted and blotted against clean paper towels. After that, 200 μl per well of secondary antibody anti-cytokine conjugate to horseradish peroxidase were added, the plate was then incubated for 2 h at room temperature. Next, the plate was aspirated and washed again four times. Two hundred microliter of substrate solution were added to each well. The plate was incubated for 20 min at room temperature and finally 50 μl of stop solutions were added to each well. Plates were read at 450 nm using a microplate reader Opsys MR (Dynex Technologies, Chantilly, VA, United States).

### Statistical Analysis

All variables were assessed and subjected to a Kolmogorov–Smirnov test to determine their distribution. Based on this, the appropriate central tendency and dispersion measures for their descriptive analysis were set.

Bivariate analysis of the relevant variables and statistical inference (where applicable) were carried out via either c^2^ or exact Fisher tests. Central tendency measures were evaluated by Student’s *t*-tests or Mann–Whitney *U*-tests for continuous variables and c^2^ or exact Fisher tests for the categorical ones. Associations were performed via Pearson correlation linear models. Data analysis was carried out using SPSS version 22.

## Results

A total of 67 subjects were studied, of which 38 (57%) were female and 29 (43%) male. Median age was of 26 years with minimum and maximum values of 13–56. Demographic data are shown in **Tables 1**, **2**. A total of 32 MS patients were considered of which 21 (66%) presented familiar background of MS and 20 (63%) suffered from crystalline luxation. 10 males and 13 females showed aortic dilation with a mean dilation value of 46 ± 11 mm, whereas the non-dilated individuals showed an average value of 30 ± 2 mm. All of the MS cases showed a systemic score greater than 7 points, with a median of 10 and extreme values (7–15 points) (**Table 2**). Mitral prolapse was found in 14 patients (43.8%). Serum biomarkers for cases and controls are shown in **Table 3**.

**Table 1:** Demographic characteristics.

	Controls *n* = 35	Patients *n* = 32	*p*
Women	21 (55)	17 (45)	NS
Men	14 (48)	15 (52)	NS
Age	29 ± 9	27 ± 10	NS
Weight	63 ± 10	74 ± 13	0.0001
Size	1.62 ± 0.08	1.80 ± 0.09	0.0001
BMI	23 ± 2	23 ± 3	NS

*BMI, body mass index. Gender expressed as frequency and percentage, other variables as mean and standard deviation (SD). A value of p = 0.05 was considered statistically significant.*

**Table 2:** Diagnostic criteria and characteristics of patients with Marfan syndrome.

Number	Gender	Age	FH	Ectopia lentis	Aortic diameter (mm)	Systemic score	Total of Ghent criteria
1	M	32	Y	Y	84	15	4
2	M	29	Y	Y	39	15	4
3	W	18	Y	N	36	10	3
4	M	37	Y	Y	84	10	4
5	W	37	N	Y	41	12	3
6	W	28	Y	Y	42	15	4
7	W	36	N	Y	44	8	3
8	M	19	Y	Y	31	10	3
9	W	22	N	Y	33	15	3
10	M	24	Y	Y	49	10	4
11	M	22	Y	N	29	12	2
12	M	56	N	Y	49	10	3
13	W	54	Y	N	39	7	3
14	M	20	Y	N	32	7	2
15	W	19	N	Y	26	8	2
16	M	29	N	Y	39	13	3
17	M	28	N	Y	58	12	3
18	W	27	N	Y	31	8	2
19	M	28	Y	N	47	10	3
20	M	18	Y	N	30	7	2
21	W	23	Y	N	39	10	3
22	W	19	N	Y	31	9	2
23	W	22	Y	N	51	10	3
24	W	21	Y	Y	29	8	3
25	M	20	Y	Y	58	12	4
26	W	27	N	N	38	7	2
27	M	26	Y	N	46	10	3
28	W	23	Y	Y	39	13	4
29	W	42	Y	N	34	7	3
30	M	18	Y	N	32	7	2
31	W	24	Y	Y	37	13	4
32	W	22	N	Y	39	7	3

*M, men; W, women; FH, family History. Systemic Positive Score is a total ≥7/20 points.*

**Table 3:** Serum biomarkers.

	Controls	Patients	*p*
TNF pg/ml	0 (0–314)	0 (0–318)	NS
IL-1 pg/ml	0 (0–296)	0 (0–198)	NS
Il-6 pg/ml	0 (0–14)	0 (0–58)	NS
IL-8 pg/ml	0 (0–29)	0 (0–139)	NS
IL-10 pg/ml	0 (0–14)	0 (0–19)	NS
VCAM pg/ml	39 (0–86)	32 (11–66)	0.03
ICAM pg/ml	336 (0–433)	334 (0–431)	NS
Osteopontin pg/ml	2314 (0–4523)	2518 (702–5207)	NS
L-Citrulline μMol/ml	62.65 ± 3.46	72.81 ± 4.35	0.06
${\text{NO}}_{3}^{-}/{\text{NO}}_{2}^{-}$ nM/ml	108.95 ± 12.05	170.04 ± 18.76	0.002
${\text{NO}}_{2}^{-}$ nM/ml	33.78 ± 3.41	43.95 ± 2.59	0.03

*Values expressed as median (minimum value – maximum) and mean ± standard deviation (SD). Tests to compare measures of central tendency were Student’s t and Mann–Whitney’s U. A value of p ≤ 0.05 was considered significant.*

In the flow-mediated dilation analysis, we found a basal diameter of 0.33 cm (Minimum: 0.31–Maximum: 0.50) for the controls vs. 0.35 cm (0.26–0.56) for MS patients (*p* = 0.04).

Regarding diastolic diameter after the first minute, there were no significant differences; the median for controls was of 0.37 cm (0.26–0.48) vs. 0.38 cm (0.28–0.59) in MS patients (*p* = 0.49).

Fractional change between basal diameter and diastolic diameter at 1 min was significantly different between groups with 12.5% ± 7.1 for controls and 5.3% ± 8.5 for MS subjects (*p* = 0.001, **Table 4**). However, the fractional change after 2 min did not show significant differences between cases and controls (6.2% ± 7.4 vs. 5.1% ± 6.6, *p* = 0.49, respectively). Twenty-one of the MS patients showed endothelial dysfunction and the percentage rate of change for flow-mediated dilation was 1.2% (-13 to 9.4%) for the ones with dysfunction and there were 11 cases without dysfunction. For these, the change was of 12.6% (10–23%; *p* = 0.01).

**Table 4:** Analysis of brachial artery diameters and percentage change.

	Basal diameter	Diameter at 1 min	Delta of diastolic diameter at 1 min	*p*	
Control	0.34 ± 0.05	0.38 ± 0.05	12.5% ± 7.1	0.001	Paired tests
Patients	0.36 ± 0.07	0.38 ± 0.07	6.2% ± 7.4	0.003	
*U* Mann–Whitney	*p* = 0.049	*p* = NS	*p* = 0.001		

*A value of p ≤ 0.05 was considered significant.*

The levels of ${\text{NO}}_{3}^{-}/{\text{NO}}_{2}^{-}$ and ${\text{NO}}_{2}^{-}$ in controls was 108.95 ± 12.05 and 33.78 ± 3.41 nM/ml in MS patients with 170.04 ± 18.76 and 43.95 ± 2.59 nM/ml (*p* = 0.002 and *p* = 0.03, respectively, **Table 3**). Citrulline levels in controls were of 62.65 ± 3.46 vs. 72.81 ± 4.35 μMol/ml in MS patients (*p* = 0.06, **Table 3**).

We found an inverse correlation of HDL and citrulline levels for patients with MS and endothelial dysfunction *R* = −0.50 (*p* = 0.01). Similarly, there was a correlation for cholesterol vs. citrulline levels *R* = −0.43 (*p* = 0.03), as well as a positive correlation between citrulline and ICAM, *R* = 0.54 (*p* = 0.04), osteopontin *R* = 0.33 (*p* = 0.07) and VCAM *R* = 0.33 (*p* = 0.06). There was also a significant correlation between the levels of citrulline and the diameter of the ascending aorta *R* = 0.62 (*p* = 0.04). In patients with endothelial dysfunction, there was also an inverse correlation between HDL and the dilation of the sine tubular junction, *R* = −0.46 (*p* = 0.03) and with the ascending aorta diameter *R* = −0.39 (*p* = 0.07). TG showed a direct correlation with the diameter of the aorta at the level of the sine tubular junction *R* = 0.58 (*p* = 0.006). The levels of ${\text{NO}}_{3}^{-}/{\text{NO}}_{2}^{-}$ in patients with endothelial dysfunction showed a direct correlation with IL-1. *R* = 0.55 (*p* = 0.01). In patients without endothelial dysfunction, there was a significant inverse correlation between citrulline levels and total cholesterol *R* = −0.57 (*p* = 0.03).

## Discussion

### Flow Mediated Vasodilation

Flow mediated vasodilation has been used as a tool to detect endothelial dysfunction in individuals with cardiovascular risk and to prevent macro and microvascular events (Al et al., 2001). Endothelial dysfunction in MS subjects is present even before a structural change in the vessels can be detected. Although, the two mechanisms may be thought as independent, structural alterations are associated with high levels of ONOO^−^ and with a poor response to vasodilating drugs. Thus, a cycle of inflammation and damage to the elastic fibers in the arterial vessels might be established (Pereira et al., 1999).

This work is one of the few studies in human MS subjects in which endothelial dysfunction was found in 62% and correlated to the presence of aortic dilation, in up to 45% of them. This value is higher than the one found in subjects without endothelial dysfunction (*p* = 0.01). This finding confirms what has been suggested in previous studies (Takata et al., 2014).

It is worth mentioning that not all of the patients presented flow-mediated endothelial dysfunction. In 11 cases, there was almost the same percentage change in the dilation than in the control group (12.6 vs. 12.5%), unlike the findings in the study by Wilson et al. (1999). However, we found that these MS patients with no endothelial dysfunction had a lower diameter of the aorta at the level of the annulus (ring) (24 vs. 27 mm, *p* = 0.04). They also showed a tendency to have a greater diameter in all other segments of the aorta similar to those found in patients with endothelial dysfunction. This observation is comparable to those of Takata et al. (2014).

This study showed a difference in the size of the basal diameter of the brachial artery between MS and controls. This result is relevant since the control group actually responded in the maximal hyperemia phase. This means that controls also have a change in flow-mediated vasodilation. However, this response differs from vasodilation in MS patients in that it is only present after 1 min. This result contrasts with the reports by Wilson et al. (1999).

NO measurements are quite relevant to evaluate the functional status of the endothelium. However, NO has a very short mean life, which makes it difficult to measure it in serum. For this reason, we measured the metabolites ${\text{NO}}_{3}^{-}/{\text{NO}}_{2}^{-}$ ratio and ${\text{NO}}_{2}^{-}$ (Baylis and Vallance, 1998).

The aortic tissue of MS patients has showed high levels of ONOO^−^ and activity of the iNOS (Soto et al., 2014) associated with the development of aortic aneurisms. Here, we showed a tendency to an increased citrulline level of up to seven times in the serum of subjects with endothelial dysfunction related to increased lipid levels. Citrulline is a metabolite of the NO synthases pathway. We also observed increased ${\text{NO}}_{3}^{-}/{\text{NO}}_{2}^{-}$ in MS patients independently of their endothelial dysfunction status. This indicates that structural changes in the arteries of MS patients prevent a response to NO generated by the friction forces (either by blood flow or vortices inside the lumen of the vessel) (Soto et al., 2016b). The axial pressure exerts perpendicular stress in the vessel (Syyong et al., 2009) as well as structural alterations in the endothelial cells (Chung et al., 2007a). Here, we established a correlation between the serum levels of NO metabolites and endothelial dysfunction assessed by flow-mediated vasodilation for the first time. Previous studies by our group have shown the involvement of the glutathione (GSH) system, that becomes exhausted with a high activity of glutathione reductase (GR) and a diminished activity of Glutathione-S-transferase (GST) and glutathione peroxidase (GPX), as well as an increase in lipoperoxydation, which was associated with ONOO^−^ increase (Zúñiga-Muñoz et al., 2017) in MS aortic tissue. These findings all together point to key mechanisms in the promotion of molecular and structural alterations in the thoracic aorta of MS patients.

Studies in animal models of MS have found that there is vasomotor dysfunction in the thoracic aorta, which may be associated with the accumulation of ONOO^−^ (Yang H.H. et al., 2010; Soto et al., 2016a), leading to endothelial-dependent vasodilation and vasoconstriction changes. These changes compromise smooth muscle contractility and increase vessel rigidity (Jondeau et al., 1999). The association between flow-mediated dilation and the diameter of the ascending aorta has been found to be negatively correlated in MS subjects with aortic dilation (Takata et al., 2014). Aside from static structural alterations like changes in the diameter of the aorta, there are other changes associated with endothelial dysfunction and bad prognosis. MS patients have a positive correlation between carotid pulse pressure (as a proxy for central pulse pressure, as a parameter for aortic distortion) and the diameter of the ascending aorta, independent of age and body surface, with negative correlation in healthy subjects. These findings associate flow-mediated endothelial dysfunction with both, static and dynamic structural alterations of the arterial vessels (Jondeau et al., 1999).

### Dyslipidemia Is Associated With Endothelial Dysfunction

When we evaluated dyslipidemia and its correlation with the endothelial function, we found that there is a tendency of lower HDL levels (down to 19 times less), as well as high LDL and TG levels in subjects with endothelial dysfunction than in subjects without it, and this finding is consistent with the findings of Liao et al. (1995). We also found evidence of statistically significant negative correlation between low HDL levels and increase of the aortic diameter in the sino-tubular segment. This dilation was positively correlated with increase in TG. It remains unclear whether the clinical relevance of these findings, however, could be associated with some implications of the alterations in the synthesis and bioavailability of NO during endothelial dysfunction and dyslipidemia. Saturated fat inhibits the production of NO, whereas polyunsaturated fat favors it, by mechanisms yet unknown. In contrast with polyunsaturated fatty acids, oleic acid inhibits the activity of eNOS, leading to lower synthesis of NO by this pathway. However, it may increase the activity of iNOS, thus favoring NO synthesis (Soto et al., 2016a). Indeed, NO formed by this pathway increases inflammation. It has been reported that MS is associated with oxidative stress and the presence of this state, may favor the oxidation of NO to ONOO^−^, leading to an increase of metalloproteinases, TGFβ and the degradation of the elastic fibers which in turn, favor the development of aortic aneurisms and rupture via a negative feedback mechanism (Yang W.I. et al., 2010). The negative effect of hypercholesterolemia over NO synthesis and endothelial dysfunction is well-documented. Oxidized LDL may act via a multitude of mechanisms, namely, inhibition of arginine transport from the blood plasma to the vascular endothelium, lowering of eNOS synthesis, interference with intracellular trafficking of eNOS from the endoplasmic reticulum to the membrane caveolae, as well as an increase in the intracellular asymmetric dimethyl arginine ADMA concentration and lowering the levels of reduced coenzyme BH_4_ (Liao et al., 1995).

Also, the increase in the intra endothelial concentrations of cholesterol favors the synthesis of caveoline-l. This protein binds eNOS forming an inactive complex. These processes constitute the basis for the biological foundation for the positive effect of statins over the endothelial function (Koh et al., 2004). We found a moderate correlation between citrulline and LDL and TG that showed a trend to increase 8 and 19-times higher in subjects with MS and endothelial dysfunction, respectively. A moderate correlation (*R* = 0.5) was also found in relation to HDL. Here, the lesser HDL, the greater citrulline and ${\text{NO}}_{3}^{-}/{\text{NO}}_{2}^{-}$ levels. Also during the inflammatory process, cell adhesion molecules are involved in the initiation and progression of atherosclerosis, as pro-inflammatory and pro-atherogenic proteins (Galkina and Ley, 2007). Furthermore was evaluated, the association of the intracellular adhesion molecules ICAM-1 and VCAM-1 with other biomarkers. In spite of not having found statistically significant differences, between cases and controls subjects or between patients with and without endothelial dysfunction, there was a moderate correlation in the patients with endothelial dysfunction and citrulline. We might explain these facts by the presence of a number of mediators, like inflammatory cytokines, TNFα, growth factors TGF-1β, free fatty acids, advanced non-enzymatic glycosylation products, LDL and angiotensin 1 (AT1), that act by stimulating their receptors at the cellular membrane. For instance, AT1 stimulation by angiotensin II promotes the synthesis of phospholipases C and D, leading to the formation of diacylglycerol and inositol triphosphate (Abe and Berk, 1998). Then, the Ca^2+^ release that these mediators provoke, leads to the activation of protein kinase C that in turn stimulates the NADPH oxidase enzyme complex, generating the formation of reactive oxygen species (ROS). ROS activate nuclear factor NF-KB allowing the expression of pro-inflammatory genes such as cytokines and chemokines like the monocyte chemotactic factor-1 (Hulsmans and Holvoet, 2010). Once activated, these mediators lead to the expression of cell adhesion molecules such as ICAM-1, VCAM-1, and E selectin at the level of the endothelial surface (Bedard and Krause, 2007).

Reactive oxygen species formation is also able to activate protein phosphorylation processes, diminishing dephosphorylation, inhibiting tyrosinphosphatase activity and the favoring the formation of mitogen activated protein kinases that are also able to activate NF-KB (Guzik and Harrison, 2007). ROS also oxidate LDL, thus augmenting their atherogenic potential and residence time at the vascular intima by binding to proteoglycans. HDL oxidation driven by ROS diminishes their anti-inflammatory properties and reverses cholesterol transport capacities (Ragbir and Farmer, 2010). Furthermore, ROS molecules inactive NO by binding and decoupling the eNOS as observed in endothelial dysfunction, but it can also increase the oxidation of NO favoring the development of ONOO^−^ (Gryglewski et al., 1986). Given our results, we have reasons to believe that this interaction represents the more prevalent mechanism behind endothelial dysfunction (Guzik et al., 2002). In addition, ROS coming from macrophages localized in the vascular intima promote the activation of MMPs leading to the degradation of the collagen capsule and rupture of the plaque (when it exists). The role that FBN-1 mutation plays and the factors and mechanisms associated with damage leading to MS are still to be completely unveiled by studying animal models and in clinical studies on human subjects (Comeglio et al., 2007).

Currently, we have evidence to believe that FBN-1, TGB-1, and TGB-2 are key starting points to the processes defining the MS phenotype. This, along with changes in the extracellular matrix and interaction with the signaling molecules just described, have provided some clues that, still need to be complemented with clinical and translational studies, allowing convergence of the clinician and basic scientists’ views on the disease triggering mechanisms.

For instance, it is well-known that other mechanisms contributing to aortic damage in MS patients, like oxidative stress and lipid deregulation, show a correlation with endothelial dysfunction (Zúñiga-Muñoz et al., 2017). These (basic) findings could be used to redesign medical management of the patients, leading to improved prognosis and evolution. Patients could profit from timely anti-oxidant, statin medication as well as nutritional and exercise plans evaluated by means of clinical assays. It is highly relevant to study tissues. We consider that endothelial dysfunction is one of the first manifestations of vascular disease. Endothelial cells have gene expression that leads to alterations in the synthesis and processing of a highly regulated protein, which show a correlation with specific mechanic/hemodynamic physicochemical processes leading to adaptive responses (Davies and Tripathi, 1993). These responses give as a result important changes in the shape, orientation and organization of endothelial cells (Reidy and Langille, 1980), as well as in changes in the ionic response to flow variations which, in turn, decrease eNOS and NO expression that generate flow-mediated vasodilation (Sumpio et al., 1988). Several factors modify these functions at the vascular endothelium, leading to endothelial dysfunction, and to disequilibrium on the bioavailability of active substances of endothelial origin predisposing to inflammation, vasoconstriction and increased vascular permeability (Badimon et al., 1992; Dejana, 1996).

## Conclusion

In MS, there is flow-mediated endothelial dysfunction, which is correlated with an increase of the aortic diameter, ${\text{NO}}_{3}^{-}/{\text{NO}}_{2}^{-}$ ratio, ${\text{NO}}_{2}^{-}$ and lipids. Endothelial dysfunction is present even before it can be detected by structural changes in the vessels and could be determined by a non-invasive technique (ultrasound). Therefore, this study suggests that this approach should be implemented during the initial diagnostic phase. Also this study, suggest that use of timely antioxidant therapy, combined with nutritional and exercise regimes, and counseling that should be evaluated by means of randomized clinical trials.

## Author Contributions

OL performed endothelial dysfunction studies and built databases. IP-T performed citrulline, ${\text{NO}}_{2}^{-}$ and ${\text{NO}}_{3}^{-}/{\text{NO}}_{2}^{-}$ assays, interpreted results, and manuscript reviser. RM performed and analyzed interleukin measurements. SC performed and analyzed computer tomography studies. AM contributed to blood analyses. CC contributed to general laboratory analyses. EH-L contributed to molecular biology discussion and manuscript writing. MS designed the study, diagnosed the patients, coordinated the general project, supervised students and directed manuscript writing. All authors read and approved the final version of the manuscript.

## Conflict of Interest Statement

The authors declare that the research was conducted in the absence of any commercial or financial relationships that could be construed as a potential conflict of interest.

## References

[B1] Abe J. Berk B. C. (1998). Reactive oxygen species as mediators of signal transduction in cardiovascular disease. *Trends Cardiovasc. Med.* 8 59–64. 10.1016/S1050-1738(97)00133-3 21235913

[B2] Al S. J. Higano S. T. Holmes D. R. Lennon R. Lerman A. (2001). Obesity is independently associated with coronary endothelial dysfunction in patients with normal or mildly diseased coronary arteries. *J. Am. Coll. Cardiol.* 37 1523–1528. 10.1016/S0735-1097(01)01212-8 11345360

[B3] Badimon L. Martínez-González J. Llorente-Cortés V. Rodríguez C. Padró T. (1992). Cell biology and lipoproteins in atherosclerosis. *Curr. Mol. Med.* 6 439–456. 10.2174/156652406778018725 16918367

[B4] Baylis C. Vallance P. (1998). Measurement of nitrite and nitrate levels in plasma and urine–what does this measure tell us about the activity of the endogenous nitric oxide system? *Curr. Opin. Nephrol. Hypertens.* 7 59–62. 10.1097/00041552-199801000-00010 9442364

[B5] Bedard K. Krause K. H. (2007). The NOX family of ROS-generating NADPH oxidases: physiology and pathophysiology. *Physiol. Rev.* 87 245–313. 10.1152/physrev.00044.2005 17237347

[B6] Chung A. W. Au Y. K. Sandor G. G. Judge D. P. Dietz H. C. van Breemen C. (2007a). Loss of elastic fiber integrity and reduction of vascular smooth muscle contraction resulting from the upregulated activities of matrix metalloproteinase-2 and -9 in the thoracic aortic aneurysm in Marfan syndrome. *Circ. Res.* 101 512–522. 17641224 10.1161/CIRCRESAHA.107.157776

[B7] Chung A. W. Yang H. H. van Breemen C. (2007b). Imbalanced synthesis of cyclooxygenase-derived thromboxane A2 and prostacyclin compromises vasomotor function of the thoracic aorta in Marfan syndrome. *Br. J. Pharmacol.* 152 305–312. 17641673 10.1038/sj.bjp.0707391 PMC2042958

[B8] Comeglio P. Johnson P. Arno G. Brice G. Evans A. Aragon-Martin J. (2007). The importance of mutation detection in Marfan syndrome and Marfan-related disorders: report of 193 FBN1 mutations. *Hum. Mutat.* 28:928. 10.1002/humu.9505 17657824

[B9] Corretti M. C. Anderson T. J. Benjamin E. J. Celermajer D. Charbonneau F. Creager M. A. (2002). Guidelines for the ultrasound assessment of endothelial-dependent flow-mediated vasodilation of the brachial artery: a report of the international brachial artery reactivity task force. *J. Am. Coll. Cardiol.* 39 257–265. 10.1016/S0735-1097(01)01746-6 11788217

[B10] Davies P. F. Tripathi S. C. (1993). Mechanical stress mechanisms and the cell. An endothelial paradigm. *Circ. Res.* 72 239–245. 10.1161/01.RES.72.2.239 8418981

[B11] Dejana E. (1996). Cell adhesion in vascular biology. *J. Clin. Invest.* 9 1949–1953. 10.1172/JCI118997 8903311 PMC507636

[B12] Dietz H. C. (2017). “Marfan syndrome,” in *GeneReviews*, eds Pagon R. A. Adam M. P. Ardinger H. H. (Seattle, WA: University of Washington), 1993–2017.

[B13] Doyle J. J. Gerber E. E. Dietz H. C. (2012). Matrix-dependent perturbation of TGFβ signaling and disease. *FEBS Lett.* 586 2003–2015. 10.1016/j.febslet.2012.05.027 22641039 PMC3426037

[B14] Eberth J. F. Taucer A. I. Wilson E. Humphrey J. D. (2009). Mechanics of carotid arteries in a mouse model of Marfan syndrome. *Ann. Biomed. Eng.* 37 1093–1104. 10.1007/s10439-009-9686-1 19350391 PMC2753508

[B15] Galkina E. Ley K. (2007). Vascular adhesion molecules in atherosclerosis. *Arterioscler. Thromb. Vasc. Biol.* 27 2292–2301. 10.1161/ATVBAHA.107.149179 17673705

[B16] Gryglewski R. J. Palmer R. M. Moncada S. (1986). Superoxide anion is involved in the breakdown of endothelium-derived vascular relaxing factor. *Nature* 320 454–456. 10.1038/320454a0 3007998

[B17] Guzik T. J. Harrison D. G. (2007). Endothelial NF-kappaB as a mediator of kidney damage: the missing link between systemic vascular and renal disease? *Circ. Res.* 101 227–229. 10.1161/CIRCRESAHA.107.158295 17673681

[B18] Guzik T. J. West N. E. Pillai R. Taggart D. P. Channon K. M. (2002). Nitric oxide modulates superoxide release and peroxynitrite formation in human blood vessels. *Hypertension* 39 1088–1094. 10.1161/01.HYP.0000018041.48432.B5 12052847

[B19] Hulsmans M. Holvoet P. (2010). The vicious circle between oxidative stress and inflammation in atherosclerosis. *J. Cell. Mol. Med.* 14 70–78. 10.1111/j.1582-4934.2009.00978.x 19968738 PMC3837590

[B20] Johnston W. F. Salmon M. Pope N. H. Meher A. Su G. Stone M. L. (2014). Inhibition of interleukin-1β decreases aneurysm formation and progression in a novel model of thoracic aortic aneurysms. *Circulation* 130(11 Suppl. 1), S51–S59. 10.1161/CIRCULATIONAHA.113.006800 25200056 PMC5097450

[B21] Jondeau G. Boutouyrie P. Lacolley P. Laloux B. Dubourg O. Laurent S. (1999). Central pulse pressure is a major determinant of ascending aorta dilation in Marfan syndrome. *Circulation* 99 2677–2681. 10.1161/01.CIR.99.20.2677 10338462

[B22] Ju X. Ijaz T. Sun H. Lejeune W. Vargas G. Shilagard T. (2014). IL-6 regulates extracellular matrix remodeling associated with aortic dilation in a fibrillin-1 hypomorphic mgR/mgR mouse model of severe Marfan syndrome. *J. Am. Heart Assoc.* 3:e000476. 10.1161/JAHA.113.000476 24449804 PMC3959679

[B23] Judge D. P. Dietz H. C. (2005). Marfan’syndrome. *Lancet* 366 1965–1976. 10.1016/S0140-6736(05)67789-6 16325700 PMC1513064

[B24] Kadoglou N. P. Papadakis I. Moulakakis K. G. Ikonomidis I. Alepaki M. Moustardas P. (2012). Arterial stiffness and novel biomarkers in patients with abdominal aortic aneurysms. *Regul. Pept.* 179 50–54. 10.1016/j.regpep.2012.08.014 22982141

[B25] Koh K. K. Ahn J. Y. Jin D. K. Han S. H. Kim H. S. Choi I. S. (2004). Comparative effects of statin and fibrate on nitric oxide bioactivity and matrix metalloproteinase in hyperlipidemia. *Int. J. Cardiol.* 97 239–244. 10.1016/j.ijcard.2003.09.007 15458690

[B26] Liao J. K. Shin W. S. Lee W. Y. Clark S. L. (1995). Oxidized low-density lipoprotein decreases the expression of endothelial nitric oxide synthase. *J. Biol. Chem.* 270 319–324. 10.1074/jbc.270.1.319 7529227

[B27] Loeys B. L. Dietz H. C. Braverman A. C. Callewaert B. L. De Backer J. Devereux R. B. (2010). The revised Ghent nosology for the Marfan syndrome. *J. Med. Genet.* 47 476–485. 10.1136/jmg.2009.072785 20591885

[B28] Pereira L. Lee S. Y. Gayraud B. Andrikopoulos K. Shapiro S. D. Bunton T. (1999). Pathogenetic sequence for aneurysm revealed in mice underexpressing fibrillin-1. *Proc. Natl. Acad. Sci. U.S.A.* 30 3819–3823. 10.1073/pnas.96.7.3819 PMC22378 10097121

[B29] Pérez-Torres I. Torres-Narváez J. C. Pedraza-Chaverri J. Rubio-Ruiz M. E. Díaz-Díaz E. Del Valle-Mondragón L. (2016). Effect of the aged garlic extract on cardiovascular function in metabolic syndrome rats. *Molecules* 21:E1425. 10.3390/molecules21111425 27792195 PMC6273338

[B30] Ragbir S. Farmer J. A. (2010). Dysfunctional high-density lipoprotein and atherosclerosis. *Curr. Atheroscler. Rep.* 12 343–348. 10.1007/s11883-010-0091-x 20506005

[B31] Reidy M. A. Langille B. L. (1980). The effect of local blood flow patterns on endothelial cell morphology. *Exp. Mol. Pathol.* 32 276–289. 10.1016/0014-4800(80)90061-1 7379981

[B32] Soto M. E. Guarner-Lans V. Herrera-Morales K. Y. Pérez-Torres I. (2018). Participation of arachidonic acid metabolism in the aortic aneurysm formation in patients with marfan syndrome. *Front. Physiol.* 9:77 10.3389/fphys.2018.00077 PMC5816394 29483877

[B33] Soto M. E. Iturriaga A. V. Guarner-Lans V. Zuñiga-Muñoz A. Aranda F. A. Velázquez E. R. (2016a). Participation of oleic acid in the formation of the aortic aneurysm in Marfan syndrome patients. *Prostaglandins Other Lipid Mediat.* 123 46–55. 10.1016/j.prostaglandins.2016.05.001 27163200

[B34] Soto M. E. Zuñiga-Muñoz A. Guarner-Lans V. Duran-Hernández E. J. Pérez-Torres I. (2016b). Infusion of *Hibiscus sabdariffa* L. modulates oxidative stress in patients with Marfan syndrome. *Mediat. Inflamm.* 2016:8625203. 10.1155/2016/8625203 27413258 PMC4927999

[B35] Soto M. E. Soria-Castro E. Lans V. G. Ontiveros E. M. Mejía B. I. Hernandez H. J. (2014). Analysis of oxidative stress enzymes and structural and functional proteins on human aortic tissue from different aortopathies. *Oxid. Med. Cell. Longev.* 2014:760694. 10.1155/2014/760694 25101153 PMC4102031

[B36] Sumpio B. E. Banes A. J. Buckley M. Johnson G. (1988). Alterations in aortic endothelial cell morphology and cytoskeletal protein synthesis during cyclic tensional deformation. *J. Vasc. Surg.* 7 130–138. 10.1016/0741-5214(88)90386-2 3336119

[B37] Syyong H. T. Chung A. W. Yang H. H. van Breemen C. (2009). Dysfunction of endothelial and smooth muscle cells in small arteries of a mouse model of Marfan syndrome. *Br. J. Pharmacol.* 158 1597–1608. 10.1111/j.1476-5381.2009.00439.x 19814726 PMC2795226

[B38] Takata M. Amiya E. Watanabe M. Omori K. Imai Y. Fujita D. (2014). Impairment of flow-mediated dilation correlates with aortic dilation in patients with Marfan syndrome. *Heart Vessels* 29 478–485. 10.1007/s00380-013-0393-3 23852405

[B39] Wilson D. G. Bellamy M. F. Ramsey M. W. Goodfellow J. Brownlee M. Davies S. (1999). Endothelial function in Marfan syndrome: selective impairment of flow-mediated vasodilation. *Circulation* 99 909–915. 10.1161/01.CIR.99.7.909 10027814

[B40] Yang H. H. van Breemen C. Chung A. W. (2010). Vasomotor dysfunction in the thoracic aorta of Marfan syndrome is associated with accumulation of oxidative stress. *Vasc. Pharmacol.* 52 37–45. 10.1016/j.vph.2009.10.005 19879959

[B41] Yang W. I. Shim C. Y. Cho I. J. Chang H. J. Choi D. Jang Y. (2010). Dyssynchronous systolic expansion of carotid artery in patients with Marfan syndrome. *J. Am. Soc. Echocardiogr.* 23 1310–1316. 10.1016/j.echo.2010.08.022 20880668

[B42] Zúñiga-Muñoz A. M. Pérez-Torres I. Guarner-Lans V. Núñez-Garrido E. Velázquez E. R. Huesca-Gómez C. (2017). Glutathione system participation in thoracic aneurysms from patients with Marfan syndrome. *Vasa* 46 177–186. 10.1024/0301-1526/a000609 28173744

